# The efficacy of a whole foods, plant-based dietary lifestyle intervention for the treatment of peripheral neuropathic pain in leprosy: a randomized control trial protocol

**DOI:** 10.3389/fnut.2023.1196470

**Published:** 2023-07-04

**Authors:** Michael Klowak, Andrea K. Boggild

**Affiliations:** ^1^Institute of Medical Science, University of Toronto, Toronto, ON, Canada; ^2^Tropical Disease Unit, Toronto General Hospital, Toronto, ON, Canada; ^3^Department of Medicine, University of Toronto, Toronto, ON, Canada

**Keywords:** leprosy, neglected tropical disease, peripheral neuropathic pain, nutrition, whole foods plant-based diet, portfolio diet, randomized control trial protocol

## Abstract

**Introduction:**

Despite effective treatment of leprosy via WHO-approved multi-drug therapy (MDT), patients still suffer from debilitating neuropathic sequelae, including peripheral neuropathic pain (PNP), and continue to develop intercurrent etiologies (such as diabetes), and progressive existing neuropathy over time. Strategies seeking to improve physiological and metabolic wellness, including those that reduce systemic inflammation and enhance immune responsiveness to neurotoxic factors may influence underlying neuropathic etiologies. A whole food plant-based diet (WFPBD) has been shown to be effective in the management of neuropathic pain due to diabetes, limiting severity and relevant symptomology. Diabetes remains a significant sequela of leprosy, as up to 50% of patients in reaction requiring corticosteroids, may develop a biochemical diabetes. As nutritional interventions may modulate both leprosy and diabetes, a specific exploration of these relationships remains relevant.

**Objectives:**

(1) To demonstrate the effect of a WFPBD lifestyle intervention, on neuropathic pain variables in leprosy; and (2) To contextualize the significance of diet in the treatment of chronic sequelae in leprosy by evaluating tolerability and side effect profile.

**Methods:**

A prospective, randomized, controlled, single-blind, multicentre interventional trial is described. Weekly one-hour dietary counseling sessions promoting a WFPBD emphasizing vegetables, fruits, whole-grains, nuts, and legumes, omitting animal products, and limiting fat intake over a six-month duration will be implemented. Participants will be 70 age and sex-matched individuals experiencing active or treated “cured” leprosy and PNP, randomized to either intervention or control groups. Primary outcome measures include efficacy via visual analog scale, subjective questionnaire and objective quantitative sensory testing, as well as safety, tolerability, and harms of a WFPBD on PNP in leprosy. This study will be initiated after Research Ethics Board (REB) approval at all participating sites, and in advance of study initiation, the trial will be registered at ClinicalTrials.gov.

**Expected impact:**

It is hypothesized that WFPBDs will mitigate progression and severity of PNP and potentially reduce the adverse events related to standard corticosteroid treatment of leprosy reactions, thereby reducing disease severity. By examining the effects of WFPBDs on PNP in leprosy, we hope to illuminate data that will lead to the enhanced therapeutic management of this neglected tropical disease.

## 1. Introduction

Leprosy continues to affect nearly 200,000 individuals globally, despite being eliminated as a public health concern by the World Health Organization in 2000 (1). Causative agents, *Mycobacterium leprae* and *Mycobacterium lepromatosis*, prevail in low-middle income countries where many barriers to treatment adherence and effective prevention of neuropathic sequelae continue to challenge the clinical management of leprosy (1, 2). Patients experience hypopigmented and inflammatory cutaneous lesions, disabling sensory and motor neuropathies, and debilitating neuropathic pain, often leading to stigma and social ostracization (2–6). Despite the development of effective therapeutics via multidrug therapy (MDT), nerve release surgery, and other immunosuppressives decades ago, treatment of the associated neuropathic pain continues to remain largely ineffective. Standard pharmacological treatment of PNP using antidepressants, anticonvulsants, and opioids, results in a less than 30% reduction of pain (1, 7). Additionally, a significant side effect profile including anticholinergic effects, dizziness, confusion, hypertension, and weight fluctuation, contribute to poor adherence to PNP treatment overall (8). Given these extensive barriers, patients with leprosy sequelae, including PNP, continue to experience a reduced quality of life even with adequate access to gold standard therapeutics (6, 9, 10). In the absence of effective pharmaceuticals, alternative interventions must be explored to bolster patients’ control of leprosy sequelae and the associated morbidity.

Lifestyle interventions have recently emerged as accessible and cost-effective strategies to reduce the burden and severity of neuropathic pain in diabetes. Strategies seeking to improve physiological and metabolic wellness, including those that reduce inflammation and enhance immune responsiveness to neurotoxic factors may influence underlying neuropathic etiologies. Nutrient supplementation has been instrumental in reducing host oxidative stress, strengthening the immune system, and mitigating potential adverse events in both leprosy and diabetes (11–15). Specifically, oral vitamin E and zinc supplementation may confer antioxidant effects, reducing daily analgesic requirements, and decreasing the incidence of leprosy symptoms overall (14, 16, 17). Likewise, studies assessing the use of oral vitamin D3 and omega-3 supplementation in diabetes have shown statistically significant improvements on subjective tests of neuropathy and pain, as well as objective tests of nerve health (11–15). Dietary interventions have also been specifically shown to reduce overall symptomology and improve the quality of life of individuals suffering from PNP due to diabetes (18–22). Many leprosy patients will develop impaired glucose tolerance due to genetic and lifestyle factors alone, and also due to the reliance on corticosteroids for control of inflammatory leprosy reactions (which can occur before, during, and after effective MDT). As patients experiencing leprosy often endure a biochemically induced diabetes, similar rationale may be applied. Prior to a final diagnosis of leprosy, patients are statistically significantly more likely to have diabetes (up to 14.2%) or pre-diabetes (up to 50%), when compared to healthy controls. Likewise, up to 65% of patients require corticosteroid treatment following reactions, resulting in steroid-induced hyperglycemia in up to 47% of individuals who were previously glucose tolerant (23–28). Additionally, given the high population prevalence of diabetes, lifestyle and genetic factors may predispose those with leprosy to diabetic neuropathy independent of corticosteroid use.

A WFPBD emphasizing vegetables, fruits, grains, and legumes, while omitting animal products, and limiting saturated fat intake has recently been shown to reduce diabetes related PNP severity and symptomology (19, 21, 22, 29, 30). In a cohort study assessing a WFPBD in 21 participants with type 2 diabetes, a statistically significant reduction of both serum triglycerides and total cholesterol, alongside a noticeable decrease in pain, weight, and insulin requirements was reported (22). Likewise, data from over 110 observational and clinical trials suggest that dietary patterns similar to a WFPBD are associated with a decreased presence of diabetes and significant improvements in biochemical profiles, including total LDL, and therapeutic requirements, for insulin in particular (21). Finally, a comprehensive randomized control trial seeking to address neuropathic pain management in 34 participants with neuropathy due to diabetes corroborates previous findings. Using both subjective questionnaire and objective quantitative sensory testing (QST), and electrophysiology, a significant decline in weight, body mass index (BMI), and glycemia was observed alongside a statistically significant improvement in neuropathic pain (19). Overall, WFPBDs have proven to be effective in the management of neuropathy due to diabetes, limiting endoneural ischemia, and providing sufficient glycemic control and lipid management, which when uncontrolled seem to exacerbate neuropathic pain. As a result, this phenomenon may translate to the management of PNP in leprosy, where diabetes comorbidity is common, and research has shown that diet and lifestyle interventions may be sufficient to control such steroid-induced hyperglycemia (23–28, 30–32). A WFPBD may also provide enhanced nutritional and antioxidant intake, conferring neuro-protective effects against leprosy alone while patients are actively undergoing antimicrobial MDT.

Major dietician groups suggest that a WFPBD is efficacious and superior in most, if not all populations. Dieticians of Canada, the British Dietetic Association, and the American Academy of Nutrition and Dietetics promote WFPBDs, suggesting improvements in weight, cholesterol, and risk of cardiovascular disease and type 2 diabetes (33–35). These groups also endorse that a WFPBD meets or exceeds recommended lipid, vitamin, mineral, fiber, protein, and amino acid requirements, when sufficient caloric intake is achieved (33–35). The wholegrains, nuts, seeds, beans, and fruits and vegetables readily supplied by a WFPBD can maximize intake of thiamine, pyridoxine, cobalamin, omega-3, zinc, and vitamins A, C, and E which are crucial to neurotransmitter and myelin synthesis, action potential propagation, and neuroprotection via capturing free radicals, and reducing oxidative stress (36–39).

We hypothesize that a WFPBD will have a multipronged beneficial effect, providing sufficient nutrient intake, neuro-protective benefits, and glycemic control to enhance host control of leprosy sequelae. A comprehensive prospective, randomized, controlled, single-blind, multicentre trial examining the efficacy, safety, and tolerability of a whole food plant-based diet on PNP in leprosy will inform any association between nutritional interventions and disease severity at the population level. Through reliable and validated techniques, such as comprehensive questionnaires and objective QST, a robust data set and analysis is expected to be generated. Overall, it is hypothesized that WFPBDs will mitigate progression of PNP as well as adverse events related to standard corticosteroid treatment of reactions, resulting in decreased neuropathic pain severity. By evaluating our current knowledge regarding the effects of WFPBDs on neuropathic pain in leprosy, we hope to illuminate data that will lead to enhanced therapeutic management and control of the impact of this neglected tropical disease.

## 2. Methods and analysis

### 2.1. Study design

**Figure 1:**
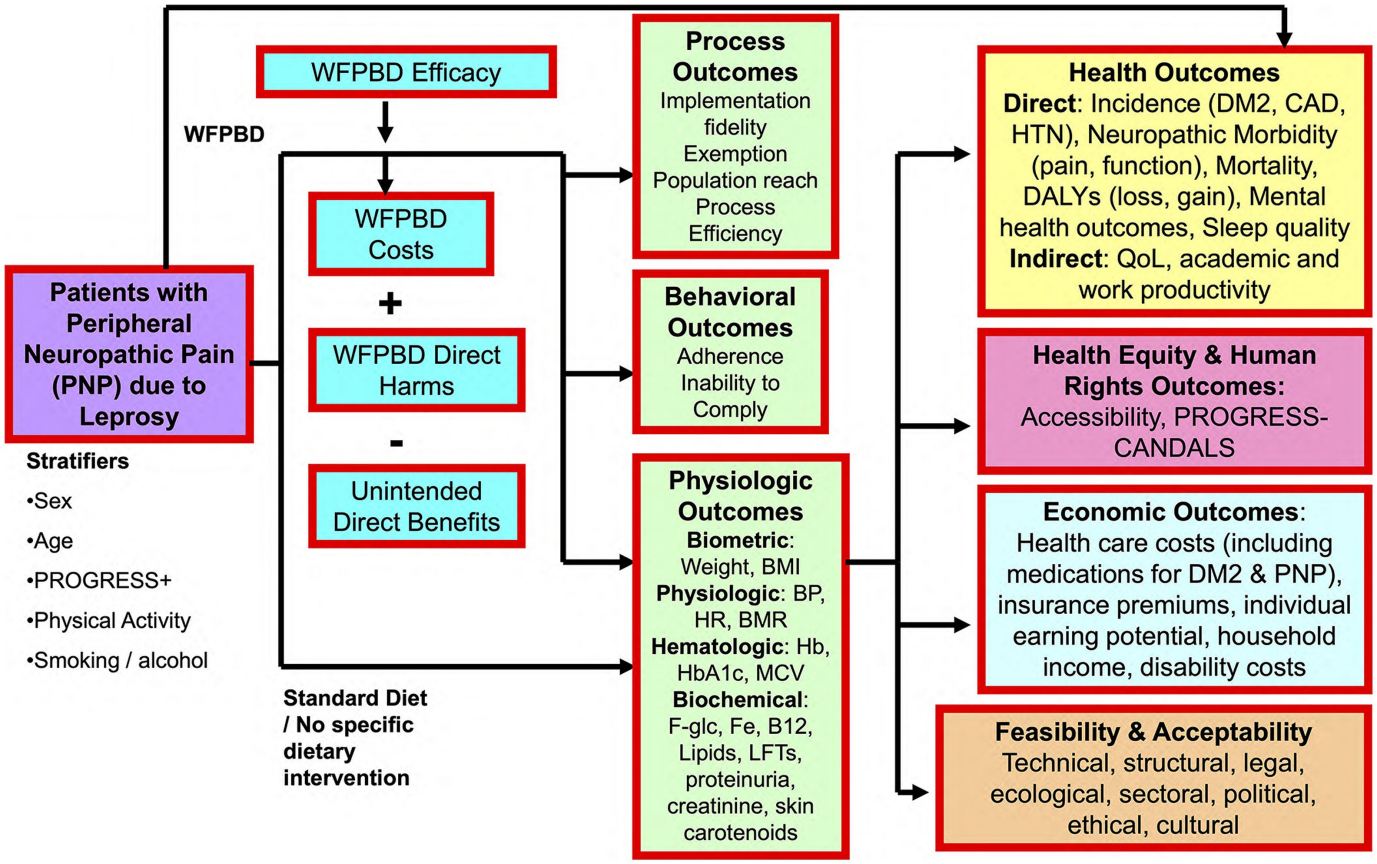
Analytic framework. BMI, body mass index; BMR, basal metabolic rate; BP, blood pressure; CAD, coronary artery disease; DALY, disability-adjusted life year; DM2, Type 2 diabetes mellitus; HR, heart rate; HTN, hypertension; LFTs, liver function tests; MCV, mean corpuscular volume; WFPBD, whole foods plant-based diet.

This is a prospective, randomized, controlled, single-blind, multicentre trial examining the efficacy, safety, and tolerability of a WFPBD versus standard diet within a population living with PNP due to leprosy. An analytic framework capturing the target population, intervention, comparator, intermediate and ultimate outcomes to be assessed, as well as the impacts and considerations underpinning such a trial is presented in Figure 1.

### 2.2. Study objectives and hypothesis

The overall objective of this study is to demonstrate the effect of a dietary lifestyle intervention, specifically a WFPBD, on neuropathic pain variables in leprosy. It is hypothesized that patients may demonstrate a statistically significant improvement in sensory and motor neuropathy, as well as pain, on comprehensive questionnaires and objective QST and muscle testing both within and between groups at study end. Secondary objectives include contextualizing the significance of diet in the treatment of chronic leprosy sequelae by demonstrating high tolerability and a limited side effect profile alongside aforementioned improvements in neuropathic pain. It is hypothesized that a WFPBD has the potential to serve as a low-risk, low-cost, low-tech intervention for chronic neuropathic pain, improving function and reducing overall morbidity of leprosy.

### 2.3. Inclusion and exclusion criteria

Participants will be enrolled at the Tropical Disease Unit (TDU) in Toronto, Ontario, Canada, and one or more partner tropical medicine institutes, and will include patients experiencing active, or treated “cured” leprosy, as well as any degree of PNP. Inclusion into the trial requires: (1) clinically compatible leprosy based on the presence of thickened peripheral nerves (on physical exam, as well as ultrasound), and hypo- or anesthetic skin lesions; and/or (2) laboratory confirmed leprosy via positive slit skin smears and/or consistent histopathology and/or the presence of acid-fast bacilli on histopathology; and/or (3) a clinically compatible presence of neuropathy on physical exam including but not limited to glove and stocking paresthesia, abnormal touch perception sensations, and subjective pain (40–42) (Table 1). Sensory perception will be evaluated using 0.2–0.6 g Semmes-Weinstein monofilament, and the presence of 2/3 insensate sites will be considered “abnormal” (43). The use of objective QST as a standalone test for inclusion will be dependent on enrolment frequency to better preserve trial specificity. In the event of inadequate enrolment, a negative QST will not exclude patients from the trial to ensure adequate sensitivity, and vice versa. QST unreliably detects small fiber neuropathy, potentially representing a large portion of the target population (44, 45). Therefore, it is unlikely that it will remain a standalone inclusion criterion, however, will still be collected for downstream analysis. Finally, prospective participants who are pregnant, <18 years of age, already following a WFPBD (including vegetarian and vegan diets), and those experiencing neuropathy due to other significant causes alone, will be excluded (Table 1). Leprosy patients who fulfill inclusion criteria experiencing a biochemical diabetes due to corticosteroid therapy will remain eligible for inclusion and will be considered in downstream analysis (23–28). No restriction on language will be implemented as translation services will be made available across study centres.

**Table 1:** Inclusion and exclusion criteria.

Inclusion	Exclusion
Presence of leprosy	Pregnancy
Clinical compatibility:	<18 years old
Thickened peripheral nerve	Already following a whole food plant-based diet
Presence of skin lesions	
Laboratory confirmation:	Experiencing neuropathy due to other significant underlying causes^†^
Positive slit skin smears	
Acid-fast bacilli on histopathology	No restriction on language as translation services will be provided
Presence of neuropathy	
Clinical compatibility on physical exam*:	
Glove and stocking paresthesia	
Abnormal touch perception sensations	
Subjective presence of pain	

*Including but not limited to; ^†^Excluding biochemically induced diabetes.

### 2.4. Dietary intervention

#### 2.4.1. Protocol diet and supporting evidence

The David Jenkins Portfolio Diet was originally designed to lower blood lipid levels in patients with hypercholesterolemia by emphasizing vegetables, fruits, grains, and legumes, while omitting animal products, and limiting saturated fat intake (46, 47). Specifically, the diet focuses on plant sterols (1 g/1000 kcals) as enriched margarine, soy and vegetable proteins (23 g/1000 kcals) as soy milk, meat alternatives, nuts, beans, chickpeas, and lentils, viscous fibers (9 g/1000 kcal) in the form of oats, barley, and psyllium, and low-calorie fruits and vegetables (46, 47) (Table 2). Following a comprehensive interventional trial in which 13 participants received the portfolio diet, a statistically significant reduction of LDL cholesterol, and coronary artery disease was observed (*p* < 0.001) alongside high compliance (93%) and acceptability (*p* = 0.042) (47). Similarly, in recent literature interventional trials have emerged assessing the efficacy of a WFPBD, like that of the portfolio diet, for neuropathic pain in individuals with diabetes. Specifically, in a trial assessing a low-fat plant-based diet, omitting animal products, and limiting fat intake to 20–30 g/day in 34 patients, a statistically significant reduction of pain on subjective neuropathy questionnaires and neuropathy on objective electrophysiology was observed (*p* < 0.05) (19). WFPBDs have been shown to provide sufficient glycemic control and lipid management which, when left unchecked, can exacerbate diabetes-related neuropathy. Given the absence of literature concerning the intersection of dietary lifestyle interventions and neuropathic pain of leprosy, it is hypothesized that a WFPBD will have similar physiological and neuro-protective benefits in a cohort of patients with leprosy and its neuropathic sequelae, in whom diabetes comorbidity is frequent.

**Table 2:** Example portfolio diet for one day.

Breakfast		Lunch		Dinner		Snacks	
35 g	Oatbran	65 g	Vegetable chili	295 g	Vegetable curry	14 g	Almond × 2
150 g	Orange	67 g	High fiber bread	85 g	Soy burger	250 g	Soymilk × 2
7 g	Metamucil	17 g	Margarine	80 g	Beans	7 g	Metamucil × 2
33 g	High fiber bread	62 g	Soy deli slices	35 g	Barleys	175 g	Soy yogurt
8 g	Margarine	80 g	Tomato	100 g	Okra	10 g	Jam
18 g	Jam	150 g	Orange	200 g	Eggplant		
250 g	Soy milk			200 g	Cauliflower		
				80 g	Onions		
				60 g	Red pepper		

Adapted from “A dietary portfolio approach to cholesterol reduction: combined effects of plant sterols, vegetable proteins, and viscous fibers in hypercholesterolemia.” by Jenkins et al. (47).

#### 2.4.2. Lifestyle counseling

Participants in the intervention group will be instructed to follow a WFPBD for 24 weeks. The study duration was chosen as a conservative estimate of comparable lifestyle-based interventional trials that demonstrated statistically significant improvements in diabetes-related neuropathy outcomes (18, 19). The diet will emphasize vegetables, fruits, whole-grains, nuts, and legumes, while omitting animal products, and limiting fat intake (Table 2). Selection of specific plant-based components may differ across study sites in concordance with local culinary preferences and cultural practices related to food. By tailoring the diet to the unique cultural diaspora affected by leprosy, many of which already endorse plant-based components, we seek to enhance adherence and acceptability to the diet overall. Likewise, current economic projections suggest that diets seeking to reduce animal products are more affordable than current standard diets across all socioeconomic levels – particularly due to inclusion of global staples such as tubers, beans, legumes, and rice – further enhancing accessibility (48–50). Participants will receive weekly support and guidance via 1 h counseling sessions with registered dieticians or the equivalent at each participating site. Sessions will be designed to disseminate comprehensive guidelines for following the WFPBD including culturally acceptable recipes and addressing concerns or knowledge gaps regarding the intervention. Participants in both the intervention group, and control group will be instructed to continue their respective leprosy therapeutics as prescribed throughout the duration of the study. Finally, dietary adherence will be measured via monthly random 24 h dietary recalls, and participants are free to withdraw at any point during the trial if they no longer wish to participate.

### 2.5. Methodology

#### 2.5.1. Collaboration

Clinical manifestations of leprosy are highly variable, hinging on several geographic, cultural, and genetic underpinnings (2). As such, to maximize the impact of trial outputs, we seek to address the global experience of leprosy through collaboration between the TDU in Toronto, Ontario, Canada, and one or more partner centres for excellence in the care of Hansen’s disease patients abroad. Given migration patterns, selected study centres will account for endemic source countries and regions including the Philippines, the Indian sub-Continent, Southeast Asia, and South America, providing for a more representative data set (2, 51). Trial documents including patient informed consent will be prepared in multiple languages to harmonize procedures across study sites. Likewise, site leads at each respective centre will be responsible for coordinating recruitment, as well as data collection. Oversight will be maintained by a centralized lead at the TDU, who will be responsible for disseminating the randomization protocol, and ensuring overall procedural adherence across sites.

#### 2.5.2. Consent, randomization, and blinding

**Figure 2:**
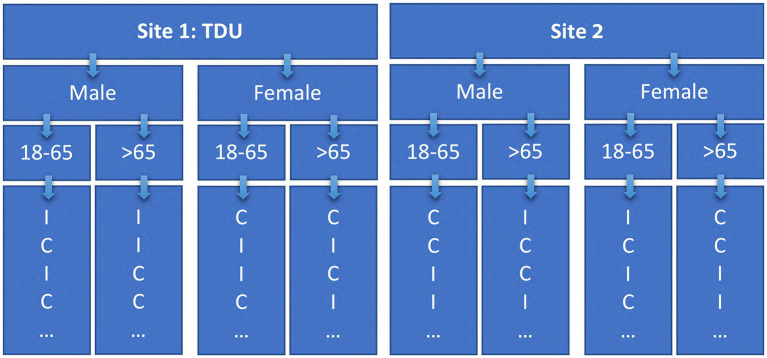
Stratified, block, randomization protocol. Adapted from: “Randomization in clinical studies.” by Lim and In (52). TDU, Tropical Disease Unit.

Patients with a history of leprosy will be pre-screened for inclusion via clinical records prior to routine follow up. Study personnel will approach eligible patients experiencing active or treated (“cured”) leprosy, as well as subjective neuropathic pain at participating tropical disease institutions. Eligible participants will receive a comprehensive in-person review of the trial protocol, including data collection requirements, and will be given the patient consent form. Patients will voluntarily confirm willingness to participate and will be free to withdraw at any point. Upon consent to enrolment, participants will be randomized to either the WFPBD intervention group, or standard of care control group. To ensure equal distribution and matching between study groups, randomization will be carried out using a stratified block method (52). Participants will be assigned to strata primarily by study site, followed by sex (male/female), and lastly age-band (18–65, >65) (Figure 2). Random sequence generation will be carried out using a computer-generated allocation sequence. Study investigators, physicians, and participants will not be blinded to group allocation due to the transparent nature of the dietary intervention and counseling. However, primary end points will be collected by independent study personnel, blinded to group allocation, allowing for a single-blinded protocol.

### 2.6. Statistical analysis

#### 2.6.1. Sample size calculation

This is a novel RCT addressing the implications of a WFPBD on neuropathic outcomes in individuals with leprosy. This relationship has not been directly assessed in the current body of literature, however similar research questions have been assessed in individuals with diabetes mellitus. As such, the sample size calculation herein will utilize this literature as a proxy. The efficacy of a WFPBD diet on neuropathy in a diabetes cohort has been reported via the Michigan Neuropathy Screening Instrument (MNSI), including both subjective (questionnaire) and objective (physical assessment) measures. The sample size calculation was based on the MNSI questionnaire measurement as explicit details pertaining to the MNSI physical assessment were not provided. Specifically, both the type of QST utilized (vibration, perception, both, etc.) and the location the test was carried out (lesion, glove, stocking, etc.) were not mentioned. Therefore, the MNSI questionnaire was preferentially chosen as a proxy. Participants receiving the intervention reported a final mean MNSI questionnaire of 5.3 ± 2.5 compared to 7.1 ± 2.8 in the control group at study end (19). Using an opensource sample size calculator with two independent study groups, continuous endpoints, an alpha of 0.5 and 80% power, a final sample size of approximately 60 participants was ascertained (53, 54). This is a conservative estimate as source literature demonstrated a statistically significant effect on outcomes with a relatively low population of 34 individuals (19). Assuming high acceptability and a 14% withdrawal and/or loss to follow-up rate based on previous literature, a final sample size of ~70 individuals, 35 per group, will be needed to adequately power this trial (19, 47, 55, 56).

#### 2.6.2. Continuous and categorical variables

Descriptive statistics including reported means ± standard deviation for continuous variables [questionnaires including MNSI-physical assessment, Neuropathy Total Symptom Score (NTSS), modified Medical Research Council Scale (mMRCS), Neuropathic Pain Symptom Inventory (NPSI), McGill Pain Questionnaire (MPQ), Pittsburgh Sleep Quality Index (PSQI), and International Physical Activity Questionnaire (IPAQ) summative scores, visual analog scale (VAS), anthropometrics, diabetes duration, hemoglobin A1c, and serum micronutrient levels] and proportions for categorical variables [number of adverse events, dietary adherence, insulin use, leprosy clinical status (according to the WHO and Ridley Jopling classification systems), frequency and prevalence of inflammatory leprosy reactions, corticosteroid use] will be reported by each study group (57). Between- and within-group comparisons will also be made for continuous variables using the Student’s *t*-test with reported *p*-values, and for categorical variables using the chi square or Fisher’s exact tests, reporting *p*-values with odds ratios and 95% confidence intervals. Statistics will also be reported for all time points including baseline, and end of study. Multivariate logistic regression adjusting for covariates [such as age, sex, PROGRESS+ factors (58, 59), level of physical activity, smoking status, alcohol consumption, comorbidities, diabetes status] and potential effect modifiers (such as change in weight over time, medication de-escalation over time) will be carried out to ascertain the impact of a WFPBD on PNP in leprosy patient sub-populations and under various conditions. All statistical analysis will be carried out using GraphPad Prism 8 (GraphPad, United States).

## 3. Projected outcomes

### 3.1. Data collection

Outcomes will be collected at both baseline and at study end by dedicated personnel blinded to group allocation. Data will be housed on a password-protected and encrypted database generated on a secure drive which will be locked in the respective study centres. Prior to database entry, all patient and study data will be anonymized, and a master list linking patient identification and consecutive study numbers will be generated, secured, and housed separately to said database and to which only the study investigators will have access. Data collected for this study cannot be linked to a specific patient, and the principal investigator and study researchers will be the only individuals who have access to this information. Following final data collection, data will be pooled across study centres and analysis will be conducted within the research lab of the principal investigator.

### 3.2. Primary outcomes

#### 3.2.1. Michigan neuropathy screening instrument

**Figure 3:**
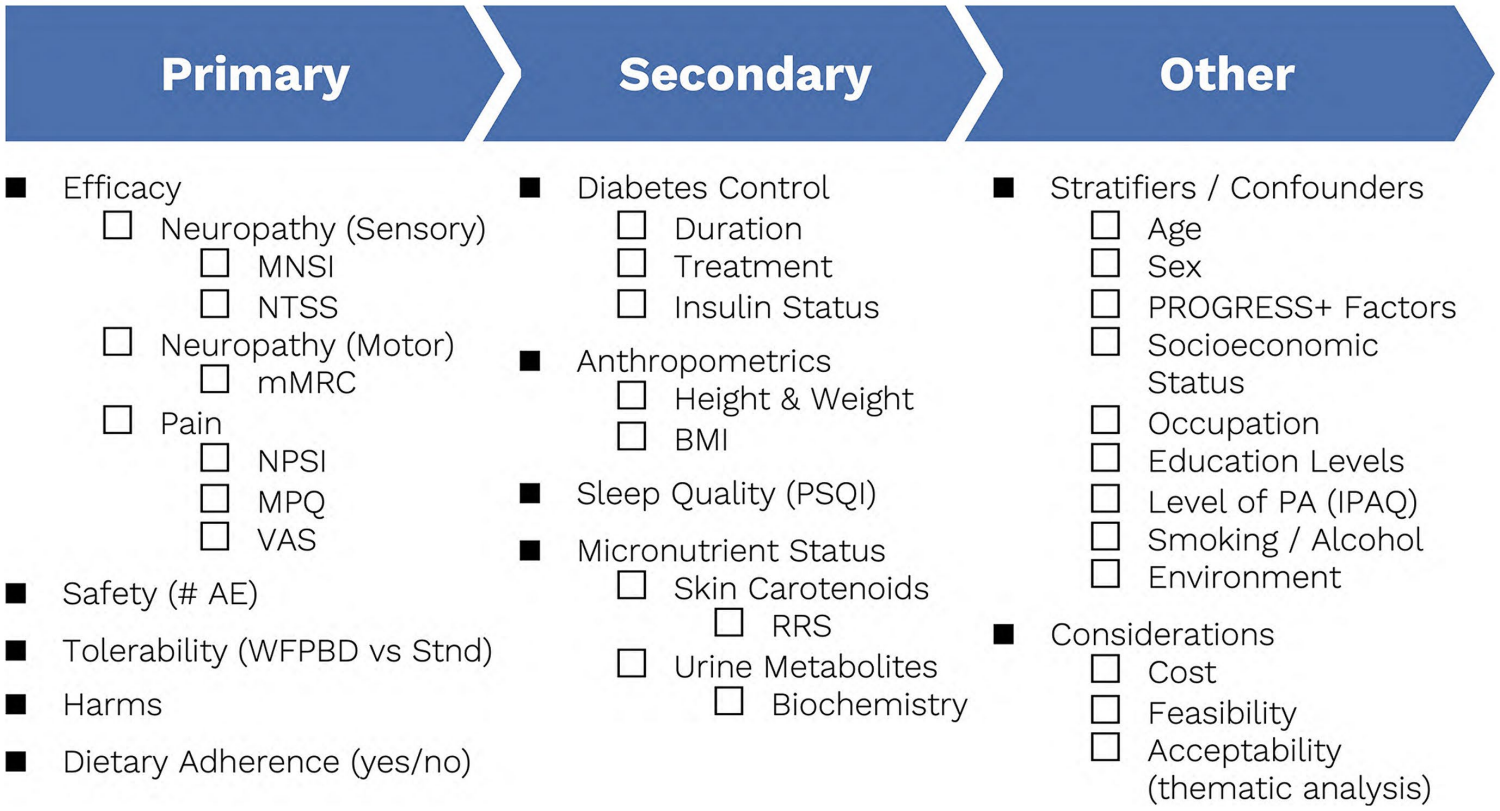
Outcomes, stratifiers, and additional considerations to be collected. AE, adverse events; BMI, body mass index; IPAQ, international physical activity questionnaire; mMRC, modified medical research council scale; MNSI, Michigan neuropathy screening instrument; MPQ, McGill pain questionnaire; NPSI, neuropathic pain symptoms inventory; NTSS, neuropathy total symptom score; PA, physical activity; PSQI, Pittsburgh sleep quality index; RRS, resonance Raman spectroscopy; Stnd, standard of care; VAS, visual analog scale; WFPBD, whole foods plant-based diet.

The MNSI is a robust assessment of neuropathy identifiers that includes both a subjective questionnaire and objective measurements of perception via QST. Specifically, Semmes-Weinstein monofilaments will be used to measure sensation at the ulnar nerve-innervated dorsum of the hand bilaterally, given this site’s predilection for leprosy associated nerve damage, while a 128 Hz tuning fork will be utilized to test for vibration perception thresholds at the great toe (43, 60). This screening instrument has been widely used and validated in both diabetes and leprosy related neuropathy cohorts (19, 60). Scoring is based on a binomial yes/no response to specific questions where yes = 1 point and no = 0 points. Additionally, any abnormal QST result = 1 point, and a score of 4 or greater indicates the presence of neuropathy (60). The MNSI will be conducted at both baseline and at study end and will be utilized as one of the measurements for sensory neuropathy (Figure 3).

#### 3.2.2. Neuropathy total symptom score

The Neuropathy Total Symptom Score will also be used to formulate a more robust assessment of sensory neuropathy. Unlike the MNSI, the NTSS includes considerations for both neuropathy intensity and frequency. Based on a summative questionnaire, participants can be assigned a maximum of 21.96 points, indicating greater morbidity, and depicted as a matrix comparing neuropathy intensity (mild, moderate, severe) versus frequency (occasional, often, continuous) (61). This questionnaire has been widely used and validated in cohorts of individuals with diabetes, a common comorbidity of leprosy, and will strengthen the assessment of sensory neuropathy within this trial (23, 61). The NTSS will be carried out at both baseline and study end (Figure 3).

#### 3.2.3. Modified medical research council scale

The modified Medical Research Council Scale is a reliable tool that has been commonly used to measure motor neuropathy in patients with leprosy (43). The mMRCS utilizes voluntary muscle testing (VMT) in the assessment of motor nerve function including the facial, ulnar, median, radial, and later popliteal nerves. Patients can be assigned a maximum of 5 points, which may be downgraded by any significant reduction of VMT. A score of less than 5 is indicative of decreased motor nerve function (43). The mMRCS will be utilized at baseline and at the end of study to provide a robust assessment of motor neuropathy in this trial (Figure 3).

#### 3.2.4. Neuropathic pain symptom inventory

The Neuropathic Pain Symptom Inventory incorporates both measures of pain intensity and frequency including common patient pain descriptors such as burning, squeezing, pressure, and stabbing relative to the past 24 h (62). It has most commonly been used in the assessment of neuropathic pain in leprosy cohorts, wherein the higher the score the greater the pain, out of a possible 100 points (62–64). Participants will complete the NPSI questionnaire as part of a comprehensive neuropathic pain assessment at both baseline and at the end of the study (Figure 3).

#### 3.2.5. McGill pain questionnaire

The McGill Pain Questionnaire will also be used to assess neuropathic pain throughout the trial, given a more robust set of patient pain descriptors. The MPQ classifies over 75 words into 4 descriptive pain categories including sensory, affective, evaluative, and miscellaneous, each with subcategories utilizing a scaled point system to indicate relative severity within each group, as well as over time. A maximum score of 78 points is possible, indicating most chronic and severe pain (65). This questionnaire has been utilized and validated within both diabetes and leprosy cohorts (19, 66). Alongside the NPSI, the MPQ will also be completed at both baseline and at the end of the study to provide the most comprehensive assessment of neuropathic pain possible (Figure 3).

#### 3.2.6. Visual analog scale

Participants will also subjectively assess pain level on a 10-cm Visual Analog Scale between absolute poles of “No pain” and “Worse possible pain,” both at baseline and at the end of the study. The VAS is a tool commonly used to assess pain given any underlying etiology, including both diabetes and leprosy, and is often utilized as the gold-standard when validating novel questionnaires assessing pain (19, 62, 63).

#### 3.2.7. Safety, tolerability, harms, adherence

Additional considerations concerning the longstanding implementation of a WFPBD within this population will also be assessed at each counseling session. Data on safety via the number of adverse events, tolerability via drop-out rate, and emerging harms, against gold standard PNP therapeutics (or non-interventions, such as watchful waiting) will be collected and summarized. Dietary adherence reported as a proportionate yes/no will also be collected to ensure interventional feasibility. Based on the current landscape of the literature a WFPBD is expected to perform as well, if not better, than the current standard of care when considering implementation (19, 47, 55) (Figure 3).

### 3.3. Secondary outcomes

#### 3.3.1. Anthropometrics, sleep quality, physical activity, and diabetes control

As a dietary lifestyle intervention can have a drastic effect on anthropometrics, height and weight will be measured at both baseline and at the end of the study and will be used to calculate body mass index (BMI) (Figure 3). Likewise, sleep quality has been associated with neuropathy severity, and must be monitored throughout the trial. The Pittsburgh Sleep Quality index is a validated questionnaire, widely used to assess sleep perturbation due to various etiologies. Participants can be assigned a maximum of 21 points indicating severe difficulties in all areas of sleep quality (67). The PSQI will be collected at baseline and at the end of the study. Self-reported physical activity will also be collected at such intervals using the International Physical Activity Questionnaire. The IPAQ includes 27-items designed to gauge the frequency and intensity of physical activity carried out in a 7-day period, encompassing occupational, transportation, housework, and leisure activities. It has been extensively tested and validated throughout 12 countries and has previously been utilized in assessing self-reported physical activity in both diabetes and leprosy cohorts (68–70). To avoid the potentially confounding effects of additional lifestyle levers, such as sleep quality and exercise, these assessments are paramount during downstream analysis. Lastly, in individuals experiencing a biochemical diabetes due to leprosy related corticosteroids, an assessment of diabetes control including duration of illness, therapeutics used, and insulin status will also be carried out to monitor any changes throughout the trial (Figure 3). Standard biochemistry, including serum cholesterol, triglycerides, hemoglobin-A1c, serum glucose, hepatic transaminases, creatinine, and basic micronutrient panel (e.g., serum ferritin, B12) will be taken to measure diabetes parameters and general physiological status. To better ascertain the relationship between the dietary lifestyle intervention and leprosy itself, neuropathy and neuropathic pain outcomes will be stratified by secondary outputs, with specific emphasis on diabetes status, and steroid use.

#### 3.3.2. Micronutrient status

In order to avoid additional invasive blood draws in at-risk equity-deserving patient populations, alternative yet validated measures of individuals’ micronutrient status will be implemented. Both skin carotenoids and urine metabolites can be utilized to define the baseline dietary habits of all participants. Resonance Raman Spectroscopy (RRS) has emerged as an inexpensive and non-invasive tool commonly used to measure skin carotenoid levels including alpha-carotene, beta-carotene, beta-cryptoxanthin, lycopene, lutein, and zeaxanthin (71, 72). Due to the high concentrations of carotenoids in fruits and vegetables, RRS has been used to reliably estimate dietary intake of such foods (71, 72). Its performance has been assessed across multiple geographies and in patients with varied skin tones, and a statistically significant correlation was not identified (71, 72). Similarly, urine metabolites can be assessed using standard laboratory biochemistry commonly available to study centres and can be utilized to estimate the remaining components of diet (73, 74). Consumption habits of fruits, vegetables, low fiber grains, fish protein, nuts, red meat, shellfish, dietary supplements, caffeine, alcohol, and fats and oils can be estimated and corroborated using relative levels of urine metabolites including methyl glutamate, mannitol, cytosine, suberate, omega 3, tryptophan, xylitol, lysine, creatine, vitamin B2, and vitamin E (73, 74). Micronutrient status assessment will be carried out at baseline and at study end to ensure significant micronutrient derangements have not occurred (Figure 3).

### 3.4. Potential stratifiers/confounders

Beyond primary and secondary endpoints, potentially confounding variables will be collected and utilized for downstream data analysis including: (1) demographic data: age, sex, socioeconomic status [relative within country and absolute according to World Bank definitions (75)], occupation (considering level of manual work, repetitive tasks, and prolonged standing/walking/climbing), household membership (living alone vs. with family members), and education level; and (2) lifestyle-related information: level of physical activity (via the international physical activity questionnaire), tobacco and cannabis smoking and alcohol consumption habits (amount consumed/day), and environment (urban vs. rural; meters a.s.l.). Effect modifiers including weight loss over time and de-escalation of medication over time will also be considered. Considerations of intervention cost, feasibility, cultural acceptability, and adherence will also be collected and summarized in a thematic analysis (Figure 3).

## 4. Ethics, dissemination, and access

In advance of study initiation and following Research Ethics Board (REB) approval at all participating centers, this trial will be registered with ClinicalTrials.gov clinical trial database (76) according to the Interventional Trial Protocol Registration Template.^1^ Current literature suggests high acceptability, and a limited risk profile associated with a WFPBD (19, 21, 22, 47, 55, 56). As a result, a safety board will not be established, however, data on relevant safety, tolerability, and harms will still be collected for downstream analysis. Iterative updates to the trial protocol will be made available to the REB and trial updates will be populated within the ClinicalTrials.gov website as soon as they become available. Data, protocol updates, and study progress will be made available to relevant stakeholders including the site principal investigators, study personnel, and participants, and will be disseminated in the form of peer-reviewed journal publications, submitted conference abstracts/posters, presentations, and lay-language infographics.

## 5. Expected impact

This trial aims to investigate the potential neuro-protective impact of a WFPBD on sensory and motor neuropathy in leprosy, thereby strengthening the literature underpinning dietary lifestyle interventions for chronic diseases. Through comprehensive validated questionnaires and QST, a robust profile of neuropathy progression and the relative impact of a WFPBD will be ascertained. Given this assessment, this trial has the potential to inform on the development of novel guidelines to better manage the sequelae of leprosy, an infectious disease of significant global relevance. Patients experiencing a leprosy infection continue to suffer from debilitating therapeutic side effects and social ostracization, contributing to ever rising disability-adjusted life years (DALYs) lost (6, 77–79). Therefore, a WFPBD in the management of leprosy-related neuropathy may mitigate the significant impact of the disease at both the patient and economic level. A WFPBD has the potential to serve as a low-risk, low-cost, low-tech intervention for chronic neuropathic pain, improving function and reducing the overall morbidity of leprosy. More broadly, this trial will also contribute to the growing literature regarding the importance of dietary lifestyle interventions in disease management and may lead to the continued investigation of this relationship in other diseases of relevance.

## Data availability statement

The original contributions presented in the study are included in the article/supplementary material, further inquiries can be directed to the corresponding author.

## Author contributions

MK contributed to protocol design and was primarily responsible for drafting the manuscript. AB conceived the protocol and contributed to design and writing of the manuscript. All authors provided input and comments on successive drafts of the manuscript, read, and approved the final draft.

## Funding

AB was supported as a Clinician Scientist by the Department of Medicine at the University of Toronto and the University Health Network. MK was supported by the Queen Elizabeth II Graduate Scholarship in Science and Technology and Open Award from the Institute of Medical Science at the University of Toronto.

## Conflict of interest

The authors declare that the research was conducted in the absence of any commercial or financial relationships that could be construed as a potential conflict of interest.

## Publisher’s note

All claims expressed in this article are solely those of the authors and do not necessarily represent those of their affiliated organizations, or those of the publisher, the editors and the reviewers. Any product that may be evaluated in this article, or claim that may be made by its manufacturer, is not guaranteed or endorsed by the publisher.

## Figures and Tables

**Table:** 

BMI	Body Mass Index
DALYs	Disability-Adjusted Life Years
IPAQ	International Physical Activity Questionnaire
MDT	Multidrug Therapy
mMRCS	modified Medical Research Council Scale
MNSI	Michigan Neuropathy Screening Instrument
MPQ	McGill Pain Questionnaire
NPSI	Neuropathic Pain Symptom Inventory
NTSS	Neuropathy Total Symptom Score
PSQI	Pittsburgh Sleep Quality Index
QST	Quantitative Sensory Testing
REB	Research Ethics Board
RRS	Resonance Raman Spectroscopy
TDU	Tropical Disease Unit
VAS	Visual Analog Scale
VMT	Voluntary Muscle Testing
WFPBD	Whole Foods Plant Based Diet

## Footnotes

^1^https://prsinfo.clinicaltrials.gov/Interventional_Study_Protocol_Registration_Template_Jan_2018.pdf
